# Cognitive-Behavioral Differences Between Officials and Folks in China's Targeted Poverty Alleviation: An Evolutionary Game Theory Perspective

**DOI:** 10.3389/fpsyg.2022.916030

**Published:** 2022-06-29

**Authors:** Zhi Chen, Chao Yang

**Affiliations:** Institute of Economic Research, Hubei Academy of Social Sciences, Wuhan, China

**Keywords:** cognitive-behavioral differences, targeted poverty alleviation, evolutionary game theory, bounded rationality, policy factors, official-folk game

## Abstract

China has historically eliminated absolute poverty and built a comprehensive well-off society through targeted poverty alleviation, at the end of which, however, many issues are worthy of scholars' attention kept emerging. A significant one was cognitive-behavioral differences between officials and folks regarding the procedure, standards, and methods of removing the poverty hats, which formed a new social dilemma called the official-folk game. Officials did not carry out targeted poverty alleviation work in strict accordance with the criteria specified by the government. In comparison, folks who have reached poverty elimination standards were unwilling to take off their poverty hats after targeted assistance due to the fear of returning to poverty. To fully explain this social dilemma, this study analyzes the causes of cognitive-behavioral differences between officials and folks in China's targeted poverty alleviation from the perspective of evolutionary game theory. The results show that bounded rational officials and folks will eventually get caught up in the prisoner's dilemma without exogenous factors' intervention. Furthermore, the study proposes that the government establish reasonable punishment, incentives, and supervision mechanisms to guide officials and folks and eliminate their cognitive-behavioral differences by investigating the influence of exogenous policy factors on the evolutionarily stable strategy (ESS) of the official-folk game. This finding not only reveals the formation mechanism of the cognitive-behavioral differences between officials and folks and presents an effective solution at the individual level but also provides a reference for other developing countries to overcome similar social dilemmas in the process of eliminating absolute poverty.

## Introduction

As globalization develops, poverty has long been not confined to a single country or region but has become a global problem. Eliminating poverty is the mission of all humanity (Anand and Ravallion, 1993). China is the world's largest developing country and whose poverty alleviation initiatives directly affect the governance of global poverty. By 2020, China's battle against poverty has been fully victorious, which got 98.99 million rural poor people rid of poverty and removed 832 poor counties and 128,000 poor villages from the poverty list according to the current standards (Xu et al., 2021b). China has completed the historic task of eradicating absolute poverty and successfully established a comprehensive well-off society.

However, the prerequisite for a comprehensive well-off society is that poor households can achieve stable and orderly poverty alleviation. Since 2015, various parts of China, especially the 14 concentrated contiguous impoverished areas, have implemented poverty exit work in succession. This work aimed to let poor households who have reached the poverty elimination standards through targeted assistance withdraw from the Targeted Poverty Alleviation Management System (abbreviated TPAMS) (Zhang et al., 2018). Nevertheless, cognitive-behavioral differences between officials and folks had severely hindered the progress of poverty exit work, which consumed more financial, material, and human resources simultaneously. These differences are mainly reflected in the following aspects:

Grassroots poverty alleviation cadres usually strictly implement poverty exit work according to the government's poverty alleviation standards and processes. In contrast, most poor households are willing to take off poverty hats after getting assistance from the government (Zeng, 2020). To achieve the poverty alleviation goals set by superiors or excessively pursuing poverty alleviation performances or other political purposes (Mandefro, 2016), however, a few grassroots poverty alleviation cadres have carried out “Digital Poverty Alleviation” (fraud on poor households' income and welfare to reduce poverty, abbreviated DPA), which leads to poor households below the poverty alleviation standards to get rid of poverty superficially and lost the assistance they deserve. As a result, some impoverished households who meet the conditions for removing poverty hats are unwilling to sign the Poverty Alleviation Confirmation and do not admit that they have been lifted out of poverty due to distrust of utilitarian and formalist officials (Grossmann et al., 2021). Besides, the fear of canceling various assistance measures and preferential conditions and returning to poverty after removing the poverty hats may be another reason (Pan et al., 2021). The cognitive-behavioral differences between officials and folks appeared in the final step of targeted poverty alleviation—poverty exit work became the last obstacle to China's poverty alleviation cause.

Many scholars have carried out related research and believe that the fundamental reason for the cognitive-behavioral differences is the government's insufficiency in formulating poverty exit policies (Liu et al., 2018; Zhang et al., 2018; Mi et al., 2022). Therefore, they argued that the cognitive-behavioral differences could be eliminated by improving the poverty exit mechanism. Liu et al. (2020) pointed out that the exit of poverty-stricken counties should be evaluated and considered from multiple aspects such as GDP, education, medical care, and transportation. Yang (2017) further proposed the four-in-one comprehensive indicator method for the exit of impoverished counties, including poverty series, material development, cultural construction, and life comfort indicators. However, these studies only remain at the macro-county level and have not involved the cognitive behaviors of individual officials or folks.

Another reason for the differences that scholars recognize is the conflict of interests between officials and folks under information asymmetry and finite rationality (Cai et al., 2022). The bounded rational officials and folks always seek to maximize immediate self-interests, thus ignoring long-term cooperative interests (Sun et al., 2021). Therefore, the root cause of the cognitive-behavioral differences between officials and folks in the official-folk game, as well as other social dilemmas (e.g., corruption, resource overexploitation, climate inaction, vaccine hesitancy, traffic congestion, and cancer metastasis), is the conflict between immediate self-interest and long-term collective interest (Arefin et al., 2020). This conflict of interest has led to cognitive differences between officials and folks in targeted poverty alleviation, which in turn has led to the differences in behavior. Ultimately, the cognitive-behavioral differences created a social dilemma that hindered poverty alleviation. To explain these social dilemmas and achieve cooperation, many social physicists have made remarkable contributions (Li et al., 2019; Tanimoto, 2019; Shen et al., 2021). Nowak (2006) presented five rules for the evolution of cooperation as follows: kin selection, group selection, direct reciprocity, indirect reciprocity, and network reciprocity. Wang et al. (2015) proposed a generic approach to estimating the dilemma strength, which as a basis, Ito and Tanimoto (2018) examined the mechanistic differences between the five rules for eliminating dilemmas by distorting/transforming the dilemma phase plane. However, these studies focused only on symmetric binary games and did not address the asymmetric case. In fact, it has been demonstrated that asymmetries arising from environmental variation or individual differences affect the evolution of cooperative behavior between interacting individuals (Kagel et al., 1996; Bshary and Grutter, 2002). In addition, this social dilemma studied in this paper falls into this category. The disparity in power, status, and information creates asymmetry in officials' and folks' payoffs. Meanwhile, limited rationality drives officials and folks to choose the best strategy for themselves in their current self-interest. This is reflected in the willingness of a few officials to risk highly severe penalties for implementing DPA and the reluctance of some poor households who have escaped poverty to exit TPAMS. Therefore, in the dilemma of the official-folk game, officials and folks can easily betray each other to make cooperation difficult. Fortunately, evolutionary game theory provided a powerful framework to investigate cooperation dilemmas inside many real-world systems (Smith, 1982; Jian et al., 2021). Numerical simulation *via* evolutionary game theory can shed some light on the complex effect of asymmetry on cooperation (Wang et al., 2021).

Therefore, based on the interview survey materials of China's rural areas, this study establishes an asymmetric binary evolutionary game model of officials (grassroots poverty alleviation cadres) and folks (poor households), reflecting the underlying logic/reason behind the social dilemma (cognitive-behavioral differences between officials and folks). In the model, officials can choose whether to adopt a trick strategy (implementing DPA) in targeted poverty alleviation, and folks can choose whether to exit the TPAMS after getting rid of poverty. At the same time, exogenous policy factors will impact the strategic benefits of officials and folks. Bounded rational officials and folks select their strategies according to their respective strategic benefits and finally reach an evolutionary game equilibrium, a stable cognitive behavior in the poverty exit work. Since the strategies of officials and folks are endogenous evolution and the results of evolutionary equilibrium depend on policy parameters, it is possible to explore the impact of government policy factors (poverty exit mechanisms) on cognitive behaviors between officials and folks in the poverty exit work. Moreover, this study investigates how the government adjusts the poverty exit mechanisms to make officials will choose the “no trick” strategy (follow the rules) and poor households will choose the “exit” strategy (take off poverty hats). This finding reveals the formation mechanism of the cognitive-behavioral differences between officials and folks at the individual level for the first time and provides an effective solution, which indicates the direction for maximizing the effectiveness of targeted poverty alleviation. Furthermore, this finding also provides a reference for other developing countries to overcome similar social dilemmas in the process of eliminating absolute poverty.

The paper is organized as follows. In section Methodology, we give a detailed description of the methodology, including the study area, research method, and data collection. In section Analysis of Cognitive-Behavioral Differences, we develop a specific analysis of cognitive-behavioral differences between officials and folks based on our investigation results. Then, we formulate the basic assumptions in section Basic Assumptions. In section Evolutionary Game Modeling, we construct an evolutionary game model and present the results of model analysis, which show the effects of policy parameters on evolutionary cooperative behavior. In section Numerical Simulation and Discussion, to present our results more prominently, we have used MATLAB for numerical simulation. Furthermore, we also discuss the above results and give our opinion. Finally, the paper is concluded in section Conclusion and Limitations.

## Methodology

### Study Area

**Figure 1:**
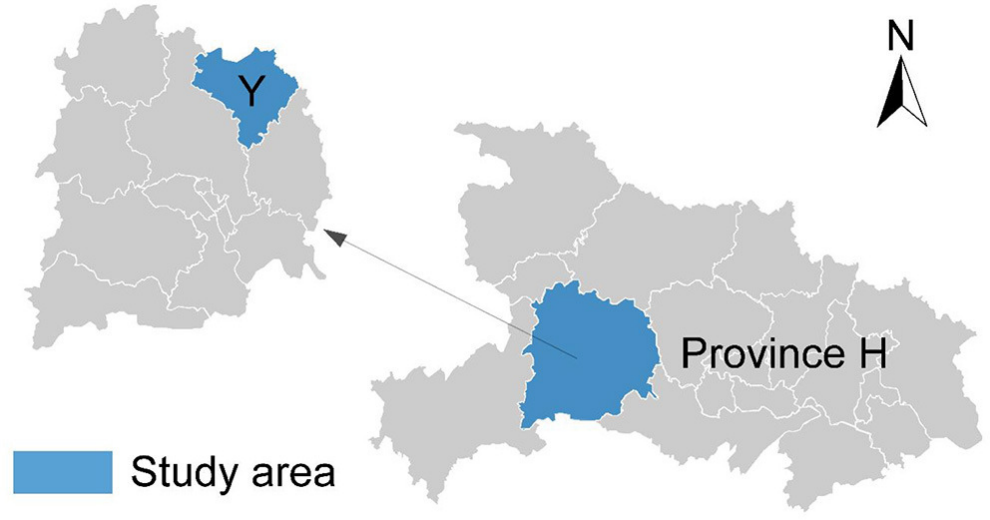
Location of the study area.

County Y, a provincial poverty-stricken county located west of Province H in Central China, is situated in the Wuling Mountains, one of the contiguous poverty-stricken areas (as shown in Figure 1). The geographical environment has various topographical landforms such as sub-alpine mountains, hills, Hegu alluvial plains, and so on., and the highest altitude of the county is 1,325 m. In terms of climate, it belongs to the subtropical continental monsoon climate, with a mild temperature and abundant rainfall. The main crops are tea, citrus, and mushrooms. In addition, the county's forest coverage area is as high as 74%, known as the “small forest sea” city. As a provincial poverty-stricken county, the number of poor people and poor villages registered in TPAMS is relatively tiny. There are 15 impoverished villages with only 24,686 people living in poverty and a 15.92% poverty incidence rate, lower than the province's average level of other impoverished counties. In addition, the population base of County Y is relatively small (only 193,700 people), which has led to the fact that the disposable income of its rural residents has been higher than the average level of all counties in Province H in the past 8 years. The good momentum of economic development has ensured that the county has quickly shifted its focus from “poverty alleviation” to “getting rid of poverty.” Since 2014, County Y has started carrying out poverty exit work and being lifted out of poverty in 2017.

### Research Method

Generally, the formal development of game theory as a theory began with the monograph “Theory of Games and Economic Behavior” by Morgenstern and Von Neumann in 1944 (Morgenstern and Von Neumann, 1953). Subsequently, Nash put forward the critical concept of “Nash Equilibrium,” which laid the theoretical foundation for non-cooperative games (Nash, 1950). After the decades of development, game theory, the core content of mainstream economics and management, has become the primary analysis tool and standard research method for scientists in almost all social science fields (Gintis, 2000).

Based on the assumption of entire rationality, Nash established a classic game theory system that still has a place in economics and management. Entire rationality requires that each game player always aims to maximize individual benefits or utility, has excellent analysis, judgment, and decision execution capabilities, and never makes mistakes. Meanwhile, entirely rationality is common knowledge of all game players. However, social people cannot be entirely rational. People may not make decisions that maximize utility due to limited information, knowledge, abilities, and unconsidered options. It is necessary to break through the assumption of entire rationality and consider all game players' bounded rationality to ensure the game analysis's practical validity. Bounded rationality considers people's fundamental physiological limitations, the resulting cognitive restrictions, motivational limitations, and their mutual influences. Bounded rationality generally has two limitations—first, incomplete information. Game players have insufficient data to list all alternatives, and there is uncertainty about the benefits of each program; second, incomplete rationality. Game players may not necessarily make decisions in an utterly self-interested manner or may not have sufficient computing power to get the optimal solution. They just choose a reasonable and satisfying answer.

Evolutionary game theory, proceeding from the assumption of bounded rationality, believes that bounded rationality first means game players often cannot or will not adopt the optimal strategy. Second, the equilibrium strategy between game players is usually caused by learning adjustments rather than a one-time choice. Third, even if the equilibrium is reached, it may deviate again. This kind of game analysis framework under bounded rationality is very similar to the biological evolution theory analysis framework, which studies the evolution and stability mechanism of the shape of biological populations based on Darwin's natural selection thought. Therefore, it is an effective way to analyze human behaviors by studying the evolution and stability mechanism of biological population traits. More importantly, the analysis method of natural evolution theory and physical behavior rules can also simulate the dynamic realization process of the game equilibrium solution. Inspired by the idea of biological evolution, Maynard Smith and Price introduced the evolutionary ideas in biological theory into game theory and published a creative paper, “The logic of animal conflict,” in 1973 and proposed the concept of evolutionary game and evolutionarily stable strategy (ESS) (Smith and Price, 1973). This kind of game analysis method originating from the theory of biological evolution is called “Evolutionary Game Theory.”

Evolutionary game theory believes that the interaction between individuals in a group is a dynamic process of the situation they face (the game environment and the state of the participants). The game situation and the participants' behaviors are interdependent. In an evolutionary game, participants' rationality constantly evolves according to the game situation changes. Rationality is a rule by which an individual chooses, and it can often be described as an individual's choice preference. As a result, bounded rationality is discussed as an individual's behavior selection rule or decision-making mechanism to determine dynamic evolutionary processes in understanding and learning the game situation. The key to evolutionary game analysis is to determine the learning mechanism of the game player and the process of strategy evolution.

### Data Collection

To deeply investigate the formation process and causes of the cognitive-behavioral differences between officials and folks in targeted poverty alleviation, our research team went to County Y, Province H, to conduct a feasibility study on poverty exit from poor counties in March 2017. First, we held a forum for poverty alleviation officials (involved county leaders and the heads of relevant departments such as the County Poverty Alleviation Office, County Easy Relocation Office, County Civil Affairs Bureau, County Human Resources and Social Security Bureau, and County Finance Bureau) at the county, township, and village levels in County Y, Province H of China, to obtain the data information and relevant supporting materials required for poverty exit.

Second, we conducted village-level (with village cadres and village task forces) interviews in five poor villages in three townships under County Y.

Third, we randomly selected 3–4 poor households in each village for household interviews. Through semistructured interviews and observations, we collected 23 valid questionnaires for officials, 19 for poor households, and other related materials in Country Y.

## Analysis of Cognitive-Behavioral Differences

The key to solving the dilemma in the poverty exit work is to make poor households lifted out of poverty willing to take off their poverty hats. However, poor households' willingness to take off poverty hats is affected by endogenous policy factors (poverty exit mechanisms) and whether grassroots poverty alleviation cadres strictly implement targeted poverty alleviation according to the government's standards and processes. In other words, cognition and behavior between grassroots poverty alleviation cadres and poor households are subjected to policy factors' macro-control. Combining semistructured interviews, field observations, and relevant literature, we will analyze cognitive-behavioral differences between officials and folks in the following.

### Officials

#### Fiscal Dilemma

The Chinese central government attached great importance to the historical task of targeted poverty alleviation and invested a lot of financial resources. In fact, due to the diminishing marginal input and output of poverty alleviation, in 2017, when the poverty incidence rate in China was <2%, this money is still a drop in the bucket for poverty alleviation of the remaining population (Liu et al., 2020). The financial difficulties encountered in targeted poverty alleviation made the grassroots government must carry out DPA.

#### Political Tournament

The tournament model is a vital incentive mechanism that refers to competition among several candidates to select winners, with relative order determining the winner (Lazear and Rosen, 1981). Companies mainly used the theory to motivate their employees to work hard, but now, it has become an important way of government governance (Su et al., 2012). The pressure-incentive political tournament adopted by the government for the officials includes administrative outsourcing, quantitative assessment, performance ranking, and merit-based promotion. The government evaluates the poverty alleviation performance of grassroots cadres every year, and the grassroots cadres who have won this performance appraisal will have a great chance of being promoted (Chen and Liu, 2011). When this tournament starts, grassroots poverty alleviation cadres become “economical people” who seek to maximize their interests and do their best to mobilize a series of financial, material, and human resources to strive for the top. As the annual tournament is held, the difficulty of getting rid of poverty will also increase year by year. Under the circumstance that the economic and political strength of the grassroots has not been greatly improved, a few grassroots cadres carrying out DPA to win the tournament have become a shortcut to the goal of poverty alleviation.

#### One-Vote Veto System

The government also formulates a target responsibility system (Mu and De Jong, 2018) for the poverty alleviation work of grassroots cadres as well as other public administration issues, which puts pressure on grassroots officials to implement their poverty alleviation responsibilities. Specifically, in the assessment at the township level, a “one-vote veto” system is implemented (Chang, 2018). As long as the poverty alleviation performance does not meet the standards, no matter how well it is done in other aspects, one vote means a year in vain. Under such severe punishment, DPA has become the helpless action of a few grassroots poverty alleviation cadres.

### Folks

#### Distrust of Officials

Although DPA is a helpless action of some grassroots cadres driven by political championships and target responsibility systems, such figures on paper are often tricky to withstand the practical inspection. In response to DPA, China's central government has formulated “Provincial Party Committee and Government Poverty Alleviation and Development Effectiveness Evaluation Measures” and established the “National Targeted Poverty Alleviation Third-Party Evaluation” organization—The State Council Poverty Alleviation Office to investigate and deal with various violations of regulations and disciplines (Liu et al., 2018). However, the grassroots government poverty alleviation cadres take various measures to get poor households rid of poverty superficially and even collude with poor households in response to the inspection of third-party assessors in the case of information asymmetry. It is manifested in the following two aspects:

##### Income Whitewash of “Two No Worries”

“Two no worries” means no worries about food and clothing (Li and Wu, 2021). In response to superior inspection, grassroots poverty alleviation cadres usually temporarily forge a subsistence allowance index, arrange poor households a public welfare post, and even increase farmers' income by temporarily participating in the industry's shareholding and dividends. However, the industry has not yet developed in most situations and is still in the planning stage.

##### Housing Whitewash of “Three Guarantees”

“Three Guarantees” means guaranteed housing, education, and medical care (Li and Wu, 2021). The adobe houses of all poor households are painted to cover up the appearance of the houses, which does not consider whether the houses meet the safety standards. For some houses that cannot be disguised by painting, grassroots poverty alleviation cadres do not provide related information to assessors or even create the illusion that the house is uninhabited or that the house owner is out with his family.

#### Dependence on Poverty Alleviation Policies

Due to the central government's determination to overcome absolute poverty, although some grassroots poverty alleviation cadres have adopted the DPA strategy, the life quality of most poor households has been significantly improved (Liu et al., 2020). Even some non-poor households conceal their actual income, such as income from working outside the home, to create the illusion of poverty for seizing benefits. Poverty alleviation policies have brought considerable benefits to poor households (almost zero cost for them), making them hugely dependent on poverty alleviation policies, which dramatically increases the difficulty of poverty exit work for grassroots poverty alleviation cadres.

#### Questions About Poverty Alleviation Standards and Process

Since 2014, grassroots poverty alleviation cadres of County Y have conducted house-to-house visits to impoverished households following the standards of “two no worries,” “three guarantees,” and income. Then, the cadres inform poor households who meet the above poverty alleviation standards and arrange for them to exit TPAMS following the steps below: confirming the poverty alleviation signature, the village committee negotiating and publicizing the announcement, reporting to the higher-level poverty alleviation department for approval and confirmation, and getting rid of poverty (Liu et al., 2020). According to this step, grassroots poverty alleviation cadres will carry out the poverty exit work as long as poor households meet the standard. However, due to the limited education level, grassroots poverty alleviation cadres feel very difficult to process the more cumbersome precision poverty alleviation forms and data files. Therefore, many work steps have been simplified, such as democratic evaluation is often only a formality, and the opinions of most villagers are not fully solicited in the process of withdrawing from object evaluation. Village committee cadres formulate the objects of poverty exit after direct discussion, and the generic structure and related information of exit poverty households have not been publicized promptly. In addition, many poor households are illiterate and have no idea about the poverty alleviation standard. Therefore, when the cadres say they have been lifted out of poverty and must sign and confirm, they still feel they have not met the poverty alleviation standard.

#### Fear of Returning to Poverty

Most people are risk-averse, and poor households are no exception. After taking off poverty hats, poor households still face the risks of returning to poverty, such as illness, incapacity to work in old age, and education costs for their children to go to school (Li et al., 2022). Out of aversion to risks and fear of returning to poverty, poor households hope that they are guaranteed by the national poverty alleviation policy forever.

In summary, the formation of cognitive-behavioral differences between officials and folks includes not only intrinsic factors (e.g., interests, concerns, and trust) but also some extrinsic factors (e.g., political, liability, and financial). These intrinsic and extrinsic factors influence the strategic choices of officials and folks in the evolutionary game. Then, their different strategic choices in their current self-interest will eventually lead to a prisoner's dilemma (i.e., grassroots poverty alleviation cadres implement DPA whereas poor households do not exit TPAMS). When the external policy factors (poverty exit mechanisms) remain the same, officials and folks will not change their own strategies. With this in mind, we present the basic assumptions of the evolutionary game in the next section.

## Basic Assumptions

### Hypothesis 1

Assume that the two populations of officials and folks are infinite and well-mixed, there are two reasons for this. First, China has a large rural population. Second, China's targeted poverty alleviation is carried out with the village as the basic unit, and many poverty alleviation cadres have been assigned.

### Hypothesis 2

Officials and folks are both bounded rational persons. Officials and folks both hope to maximize their self-interests in targeted poverty alleviation. Due to information asymmetry, however, folks do not know whether officials implemented DPA in poverty exit work, and officials also do not know whether folks will discover that.

### Hypothesis 3

The influence of policy factors (poverty exit mechanisms) on officials and poor households is an exogenous given, which is used to explore the impact of policy factors on the equilibrium of the evolutionary game.

### Hypothesis 4

The basic income earned by officials who typically complete the poverty alleviation indicators according to the government's requirements is *U*_*G*_ (including political performance, reputation, wages, etc.), and the essential cost paid is *C*_*G*1_ (including time and energy for normal work, etc.). The primary benefit of folks in targeted poverty alleviation is *U*_*P*_ (including direct and indirect subsidies). Due to the public welfare nature of the poverty alleviation policy, although folks hardly need to pay any cost in the process, the impact of various risks makes folks possibly return to poverty. Therefore, this study sets the possible loss caused by returning to poverty as η_1_*R*(*R* ≤ *U*_*P*_). The poverty regression coefficient η_1_ represents the estimation of the risk of returning to poverty. The larger η_1_ is the more negative poor households about returning to poverty. If poor households choose the “no exit” strategy (do not take off poverty hats), officials will persuade folks to quit with cost *C*_*G*3_. However, for folks who choose the “no exit” strategy, this strategy choice is their optimal consideration for the current situation in the game. Once they have made this decision, they will not change until they receive more feedback of information, so the extra efforts of officials are still useless in the short term. As a result, officials can choose the “trick” strategy (DPA) to complete the poverty alleviation target. Nevertheless, the considerable conflict caused by the “trick” strategy of officials and the “no exit” strategy of the nail households will make officials have a higher chance of being discovered by government inspectors. In this case, the promotion benefit is *P*_1_, and the penalty is δ_2_*P*_2_(δ_2_ > δ_1_). δ_2_ *and δ*_1_ indicate the magnitude of the likelihood that government inspectors will find that officials have committed DPA.

### Hypothesis 5

When officials adopt the “trick” strategy, if folks choose the “exit” strategy, then officials can make themselves pay less by DPA. This study sets this cost reduction as *C*_*G*2_. At the same time, due to the existence of the political tournament, officials who adopt the “trick” strategy can get better promotion opportunities with an income of *P*_1_. However, when officials adopt the “trick” strategy, they have a probability of δ_1_ to be found (depending on the government's supervision) with punishment *P*_2_ (including administrative penalties and reputation reduction) when they are found.

### Hypothesis 6

When officials adopt the “no trick” strategy, if folks choose the “exit” strategy, officials will get the essential benefit *U*_*G*_ and pay the basic cost *C*_*G*1_. If folks choose the “no exit” strategy, officials need to pay extra effort cost *C*_*G*3_ and punishment *P*_3_ for not fulfilling the government's poverty alleviation targets (one-vote veto system).

### Hypothesis 7

When folks choose the “exit” strategy, if officials choose the “no trick” strategy, the folks' primary benefit is *U*_*P*_, and the cost is η_1_*U*_*P*_. If officials choose the “trick” strategy, in this case, the basic income of folks will be reduced *U*_*T*_ due to the DPA of officials. The “trick” strategy of officials will make folks more pessimistic about returning to poverty with a poverty return coefficient η_2_(> η_1_).

### Hypothesis 8

When folks choose the “no exit” strategy, if officials select the “no trick” strategy, the basic income for folks will be *U*_*P*_. At the same time, becoming a nail household may reduce the evaluation of the same villagers and increase the risk of “wearing small shoes” for officials (being targeted or sidelined by officials in some ways) in the future. This study sets this part of the reputation cost as *E*(< *R*). If officials choose the “trick” strategy, basic income will reduce *U*_*T*_ due to the DPA of officials, and folks still pay reputation cost *E*.

### Hypothesis 9

Suppose that the proportion of officials who choose the “no trick” strategy is *x*(0 ≤ *x* ≤ 1), and the ratio who select the “trick” strategy is 1 − *x*. The proportion of folks who choose the “exit” strategy is *y*(0 ≤ *y* ≤ 1), and the ratio of selecting the “no exit” strategy is 1 − *y*. Both *x* and *y* are functions of time *t*.

## Evolutionary Game Modeling

### Game Payoff Matrix

According to the above analysis and assumptions, we can obtain the game payment matrix between officials and folks (as shown in Table 1).

**Table 1:** Game payment matrix between officials and folks.

		**Folks**	
		**Exit**	**No exit**
**Officials**	**No trick**	*U*_*G*_ − *C*_*G*1_, *U*_*P*_ − η_1_*R*	*U*_*G*_ − *C*_*G*1_ − *C*_*G*3_ − *P*_3_, *U*_*P*_ − *E*
	**Trick**	*U*_*G*_ − *C*_*G*1_ + *C*_*G*2_ + *P*_1_ − δ_1_*P*_2_, *U*_*P*_ − η_2_*R* − *U*_*T*_	*U*_*G*_ − *C*_*G*1_ + *C*_*G*2_ + *P*_1_ − δ_2_*P*_2_ − *C*_*G*3_, *U*_*P*_ − *E* − *U*_*T*_

### Equilibrium of the Game

#### Replicator Dynamic Equation of Officials

Set the expected utility and group utility of officials choosing the “no trick” strategy and “trick” strategy as *U*_11_, *U*_12_, *U*_1_, respectively:


(1) $$\begin{array}{l} {\text{U}}_{11}=\text{y}\left({\text{U}}_{\text{G}}-{\text{C}}_{\text{G}1}\right)+\left(1-\text{y}\right)\left({\text{U}}_{\text{G}}-{\text{C}}_{\text{G}1}-{\text{C}}_{\text{G}3}-{\text{P}}_{3}\right) \end{array}$$

(2) $$\begin{array}{l} {\text{U}}_{12}=\text{y}\left({\text{U}}_{\text{G}}-{\text{C}}_{\text{G}1}+{\text{C}}_{\text{G}2}+{\text{P}}_{1}-\delta_{1}{\text{P}}_{2}\right)+\left(1-\text{y}\right)\\ \left({\text{U}}_{\text{G}}-{\text{C}}_{\text{G}1}+{\text{C}}_{\text{G}2}+{\text{P}}_{1}-\delta_{2}{\text{P}}_{2}-{\text{C}}_{\text{G}3}\right) \end{array}$$

(3) $$\begin{array}{l} {\text{U}}_{1}=\text{x}{\text{U}}_{11}+\left(1-\text{x}\right){\text{U}}_{12} \end{array}$$

According to the Malthusian dynamic equation, when a group in the game chooses a specific strategy with a higher return than the group that selects another strategy, it is considered that the strategy can adapt to the evolution process of the group (Friedman, 1991).

**Figure 2:**
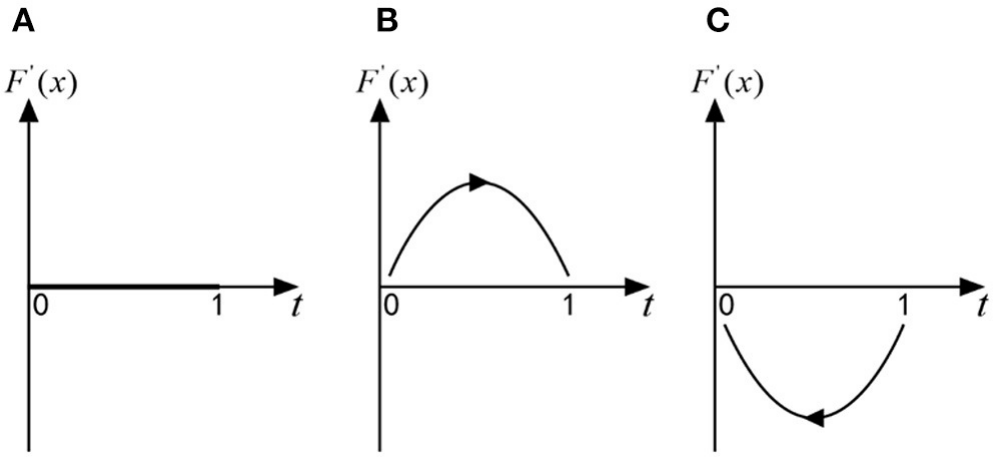
**(A–C)** The replication dynamic phase diagram of officials in the official-folk game.

The replicator dynamic equation for officials to choose the “no trick” strategy is:


$$\begin{array}{l} F\left(x\right)=\frac{dx}{dt}=x\left(U_{11}-U_{1}\right)=x\left[U_{11}-xU_{11}-\left(1-x\right)U_{12}\right]\\ \;=x\left(1-x\right)\left(U_{11}-U_{12}\right) \end{array}$$

(4) $$\begin{array}{l} =x\left(1-x\right)\left(yP_{3}+y\delta_{1}P_{2}-y\delta_{2}P_{2}+\delta_{2}P_{2}-P_{3}-C_{G2}-P_{1}\right) \end{array}$$

(5) $$\begin{array}{l} {\text{F}}^{{\;}^{\prime }}\left(\text{x}\right)=\frac{\text{dF}\left(\text{x}\right)}{\text{dx}}=\left(1-2\text{x}\right)\left(\text{y}{\text{P}}_{3}+\text{y}\delta_{1}{\text{P}}_{2}-\text{y}\delta_{2}{\text{P}}_{2}\right)\\ \left(+\delta_{2}{\text{P}}_{2}-{\text{P}}_{3}-{\text{C}}_{\text{G}2}-{\text{P}}_{1}\right) \end{array}$$

when *F*(*x*) = 0, the solution of the equation is *x* = 0; *x* = 1; $y^{*}=(P_{3}+C_{G2}+P_{1}-\delta_{2}P_{2})/(P_{3}+\delta_{1}P_{2}-\delta_{2}P_{2})(0\leq y^{*}\leq 1)$. According to the nature of the evolutionarily stable strategy of replicating dynamic equations, *F*(*x*) = 0 and *F*′(*x*) ≤ 0 must be satisfied when the evolution is stable. Then, the following discussion is conducted:

if $y=y^{{\;}^{*}}=\left(P_{3}+C_{G2}+P_{1}-\delta_{2}P_{2}\right)/\left(P_{3}+\delta_{1}P_{2}-\delta_{2}P_{2}\right)$, for any *x*, *F*(*x*) = 0, *F*′(*x*) = 0, then axis *x* is in a stable state, which shows that when folks choose the “exit” strategy with the probability of (*P*_3_ + *C*_*G*2_ + *P*_1_ − δ_2_*P*_2_)/(*P*_3_ + δ_1_*P*_2_ − δ_2_*P*_2_), any strategy of officials is an ESS. As shown in Figure 2A;

if $y>y^{{\;}^{*}}=\left(P_{3}+C_{G2}+P_{1}-\delta_{2}P_{2}\right)/\left(P_{3}+\delta_{1}P_{2}-\delta_{2}P_{2}\right)$, *F*(0) = 0, *F*′ (0) > 0 but *F*(1) = 0,*F*′(1) < 0, then *x* = 1 is the only ESS, which shows that when folks choose the “exit” strategy with a probability greater than (*P*_3_ + *C*_*G*2_ + *P*_1_ − δ_2_*P*_2_)/(*P*_3_ + δ_1_*P*_2_ − δ_2_*P*_2_), officials will shift from the “trick” strategy to the “no trick” strategy, and the “no trick” strategy is an ESS. As shown in Figure 2B;

if $y<y^{{\;}^{*}}=\left(P_{3}+C_{G2}+P_{1}-\delta_{2}P_{2}\right)/\left(P_{3}+\delta_{1}P_{2}-\delta_{2}P_{2}\right)$, *F*(0) = 0,*F*′ (0) < 0 but *F*(1) = 0,*F*′(1) > 0, then *x* = 0 is the only ESS, which shows that when folks choose the “exit” strategy with a probability less than (*P*_3_ + *C*_*G*2_ + *P*_1_ − δ_2_*P*_2_)/(*P*_3_ + δ_1_*P*_2_ − δ_2_*P*_2_), officials will shift from the “no trick” strategy to the “trick” strategy, and the “trick” strategy is an ESS. As shown in Figure 2C;

#### Replicator Dynamic Equation of Folks

Set the expected utility and group utility of folks choosing the “exit” strategy and “no exit” strategy as *U*_21_, *U*_22_, *U*_2_, respectively:


(6) $$\begin{array}{l} {\text{U}}_{21}=\text{x}\left({\text{U}}_{\text{P}}-\eta_{1}\text{R}\right)+\left(1-\text{x}\right)\left({\text{U}}_{\text{P}}-\eta_{2}\text{R}-{\text{U}}_{\text{T}}\right) \end{array}$$

(7) $$\begin{array}{l} {\text{U}}_{22}=\text{x}\left({\text{U}}_{\text{P}}-\text{E}\right)+\left(1-\text{x}\right)\left({\text{U}}_{\text{P}}-\text{E}-{\text{U}}_{\text{T}}\right) \end{array}$$

(8) $$\begin{array}{l} {\text{U}}_{2}=\text{y}{\text{U}}_{21}+\left(1-\text{y}\right){\text{U}}_{22} \end{array}$$

**Figure 3:**
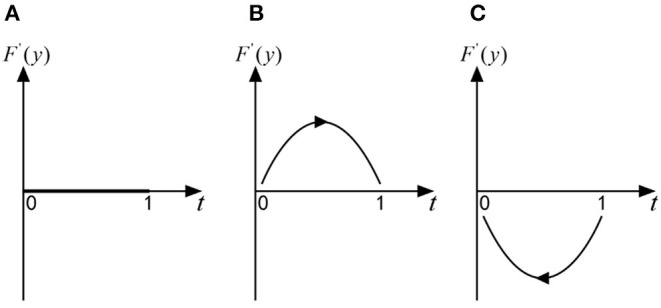
**(A–C)** The replication dynamic phase diagram of folks in the official-folk game.

The replicator dynamic equation for folks to choose the “exit” strategy is:


(9) $$\begin{array}{l} \text{F}\left(\text{y}\right)=\frac{\text{dy}}{\text{dt}}=\text{y}\left({\text{U}}_{21}-{\text{U}}_{2}\right)=\text{y}\left(1-\text{y}\right)\left({\text{U}}_{21}-{\text{U}}_{22}\right)\\ =\text{y}\left(1-\text{y}\right)\left(\eta_{2}\text{Rx}-\eta_{1}\text{Rx}-\eta_{2}\text{R}+\text{E}\right) \end{array}$$

(10) $$\begin{array}{l} {\text{F}}^{{\;}^{\prime }}\left(\text{y}\right)=\left(1-2\text{y}\right)\left(\eta_{2}\text{Rx}-\eta_{1}\text{Rx}-\eta_{2}\text{R}+\text{E}\right) \end{array}$$

when *F*(*y*) = 0, the solution of the equation is *y* = 0; *y* = 1; $x^{{\;}^{*}}=\left(\eta_{2}R-E\right)/\left[\left(\eta_{2}-\eta_{1}\right)R\right]\left(0\leq x^{{\;}^{*}}\leq 1\right)$. According to the nature of the evolutionarily stable strategy of replicating dynamic equations, *F*(*y*) = 0 and *F*′(*y*) ≤ 0 must be satisfied when the evolution is stable. Then, the following discussion is conducted:

if $x=x^{{\;}^{*}}=\left(\eta_{2}R-E\right)/\left[\left(\eta_{2}-\eta_{1}\right)R\right]$, for any *y*, *F*(*y*) = 0, *F*′(*y*) = 0, then axis *y* is in a stable state, which shows that when officials adopt the “no trick” strategy with the probability of (η_2_*R* − *E*)/[(η_2_ − η_1_)*R*], any strategy of folks is an ESS. As shown in Figure 3A;

if $x>x^{{\;}^{*}}=\left(\eta_{2}R-E\right)/\left[\left(\eta_{2}-\eta_{1}\right)R\right]$, *F*(0) = 0, *F*′ (0) > 0 but *F*(1) = 0, *F*′(1) < 0, then *y* = 1 is the only ESS, which shows that when officials adopt the “no trick” strategy with a probability greater than (η_2_*R* − *E*)/[(η_2_ − η_1_)*R*], folks will shift from the “no exit” strategy to the “exit” strategy, and the “exit” strategy is an ESS. As shown in Figure 3B;

if $x<x^{{\;}^{*}}=\left(\eta_{2}R-E\right)/\left[\left(\eta_{2}-\eta_{1}\right)R\right]$, *F*(0) = 0, *F*′ (0) < 0 but *F*(1) = 0, *F*′(1) > 0, then *y* = 0 is the only ESS, which shows that when officials adopt the “no trick” strategy with a probability less than (η_2_*R* − *E*)/[(η_2_ − η_1_)*R*], folks will shift from the “exit” strategy to the “no exit” strategy, and the “no exit” strategy is an ESS. As shown in Figure 3C;

#### ESS Analysis

On the plane *M* = {(*x, y*)|0 ≤ *x, y* ≤ 1}, there are five partial equilibrium points (0, 0),(0, 1),(1, 0),(1, 1),(x^*^, y^*^) for both sides of the game. $x^{{\;}^{*}}=\left(\eta_{2}R-E\right)/\left[\left(\eta_{2}-\eta_{1}\right)R\right]\left(0\leq x^{{\;}^{*}}\leq 1\right)$,${\;y}^{{\;}^{*}}=\left(P_{3}+C_{G2}+P_{1}-\delta_{2}P_{2}\right)/\left(P_{3}+\delta_{1}P_{2}-\delta_{2}P_{2}\right)\left(0\leq x^{{\;}^{*}},y^{{\;}^{*}}\leq \;1\right)$.

Jacobian matrix is:


$$\begin{array}{l} \text{J}=\left[\begin{array}{c} \frac{\partial \text{F}\left(\text{x}\right)}{\partial \text{x}}\;\frac{\partial \text{F}\left(\text{x}\right)}{\partial \text{y}}\\ \frac{\partial \text{F}\left(\text{y}\right)}{\partial \text{x}}\;\frac{\partial \text{F}\left(\text{y}\right)}{\partial \text{y}} \end{array}\right] \end{array}$$

(11) $$\begin{array}{l} =\left[\begin{array}{cc} \left(1-2\text{x}\right)\left(\begin{array}{c} \text{y}{\text{P}}_{3}+\text{y}\delta_{1}{\text{P}}_{2}-\text{y}\delta_{2}{\text{P}}_{2}\\ +\delta_{2}{\text{P}}_{2}-{\text{P}}_{3}-{\text{C}}_{\text{G}2}-{\text{P}}_{1} \end{array}\right) & \text{x}\left(1-\text{x}\right)\left({\text{P}}_{3}+\delta_{1}{\text{P}}_{2}-\delta_{2}{\text{P}}_{2}\right)\\ \begin{array}{c} \text{y}\left(1-\text{y}\right)\\ \left(\eta_{2}\text{R}-\eta_{1}\text{R}\right) \end{array} & \begin{array}{c} \left(1-2\text{y}\right)\\ \left(\eta_{2}\text{Rx}-\eta_{1}\text{Rx}-\eta_{2}\text{R}+\text{E}\right) \end{array} \end{array}\right] \end{array}$$

The determinant and trace of matrix *J* are, respectively:


$$\begin{array}{l} \text{d}et\text{J}=\left(1-2\text{x}\right)\left(\text{y}{\text{P}}_{3}+\text{y}\delta_{1}{\text{P}}_{2}-\text{y}\delta_{2}{\text{P}}_{2}+\delta_{2}{\text{P}}_{2}-{\text{P}}_{3}-{\text{C}}_{\text{G}2}-{\text{P}}_{1}\right) \end{array}$$

(12) $$\begin{array}{l} {}*\left(1-2\text{y}\right)\left(\eta_{2}\text{Rx}-\eta_{1}\text{Rx}-\eta_{2}\text{R}+\text{E}\right)-\text{y}\left(1-\text{y}\right)\left(\eta_{2}\text{R}-\eta_{1}\text{R}\right)\\ \text{x}\left(1-\text{x}\right)\left({\text{P}}_{3}+\delta_{1}{\text{P}}_{2}-\delta_{2}{\text{P}}_{2}\right) \end{array}$$

(13) $$\begin{array}{l} \text{t}r\text{J}=\left(1-2\text{x}\right)\left(\text{y}{\text{P}}_{3}+\text{y}\delta_{1}{\text{P}}_{2}-\text{y}\delta_{2}{\text{P}}_{2}+\delta_{2}{\text{P}}_{2}-{\text{P}}_{3}-{\text{C}}_{\text{G}}^{2}-{\text{P}}_{1}\right)\\ +\left(1-2\text{y}\right)\left(\eta_{2}\text{Rx}-\eta_{1}\text{Rx}-\eta_{2}\text{R}+\text{E}\right) \end{array}$$

According to Friedman's discriminant method (Friedman, 1998), the Jacobian matrix can analyze ESS that must satisfy *detJ* > 0 and *trJ* < 0. (x^*^, y^*^) Set *trJ* = 0, so ESS will not be obtained here. We substitute the remaining four equilibrium points into *detJ* and *trJ* to obtain Table 2.

**Table 2:** The expression of matrix determinant and trace corresponding to equilibrium points.

**(x,y)**	**Expression of matrix determinant and trace**
(0, 0)	*detJ* = (δ_2_*P*_2_ − *P*_3_ − *C*_*G*2_ − *P*_1_) (*E* − η_2_*R*)
	*trJ* = (δ_2_*P*_2_ − *P*_3_ − *C*_*G*2_ − *P*_1_) + (*E* − η_2_*R*)
(0, 1)	*detJ* = −(δ_1_*P*_2_ − *C*_*G*2_ − *P*_1_)(*E* − η_2_*R*)
	*trJ* = (δ_1_*P*_2_ − *C*_*G*2_ − *P*_1_)−(*E* − η_2_*R*)
(1, 0)	*detJ* = −(δ_2_*P*_2_ − *P*_3_ − *C*_*G*2_ − *P*_1_)*(*E* − η_1_*R*)
	*trJ* = −(δ_2_*P*_2_ − *P*_3_ − *C*_*G*2_ − *P*_1_) + (*E* − η_1_*R*)
(1, 1)	*detJ* = (δ_1_*P*_2_ − *C*_*G*2_ − *P*_1_)(*E* − η_1_*R*)
	*trJ* = −(δ_1_*P*_2_ − *C*_*G*2_ − *P*_1_)−(*E* − η_1_*R*)

For the convenience of analysis, we replace several expressions that repeatedly appear in Table 2 with letters. For details, please refer to Table 3.

**Table 3:** Meaning of letters.

**Letter**	**Expression**	**Meaning**
*A*	δ_2_*P*_2_ − *P*_3_ − *C*_*G*2_ − *P*_1_	The part of officials' “no trick” strategy benefits that exceed the “trick” strategy benefits when folks choose the “no exit” strategy
*B*	*E* − η_2_*R*	The part of folks' “exit” strategy benefits that exceed the “no exit” strategy benefits when officials choose the “trick” strategy
*C*	δ_1_*P*_2_ − *C*_*G*2_ − *P*_1_	The part of officials' “no trick” strategy benefits that exceed the “trick” strategy benefits when folks choose the “exit” strategy
*D*	*E* − η_1_*R*	The part of folks' “exit” strategy benefits that exceed the “no exit” strategy benefits when officials choose the “no trick” strategy

Different parameter changes in the game will affect the symbols of *detJ* and *trJ* at the equilibrium point, thus forming different ESS. The symbols of *detJ* and *trJ* are determined by the symbols of letters *A*, *B*, *C*, and *D* after the replacement. Therefore, according to the different combinations of *A*, *B*, *C*, and *D* symbols, this study divides ESS of officials and folks into the following eleven situations:

*A, B, C, D* > 0

There is only one ESS (1, 1) in the game, which needs to satisfy δ_2_*P*_2_ > *P*_3_ + *C*_*G*2_ + *P*_1_, δ_1_*P*_2_ > *C*_*G*2_ + *P*_1_, *E* > η_2_*R* > η_1_*R*. *A, C* > 0. This shows that regardless of whether folks choose the “exit” strategy, the benefits of officials who choose the “no trick” strategy are higher than those who choose the “trick” strategy. Under such circumstances, officials will always choose the “no trick” strategy, called the dominant strategy of officials. *B, D* > 0 means that no matter whether officials choose the “trick” strategy, the benefits of folks who choose the “exit” strategy is higher than “no exit” strategy, and the “exit” strategy is the dominant strategy of folks.

##### *A, C, D* > 0, *B* < 0

There is only one ESS (1, 1) in the game, which needs to satisfy δ_2_*P*_2_ > *P*_3_ + *C*_*G*2_ + *P*_1_, δ_1_*P*_2_ > *C*_*G*2_ + *P*_1_, η_2_*R* > *E* > η_1_*R*. Likewise, the “no trick” strategy is still the dominant strategy of officials. *B* < 0, *D* > 0 shows that if officials choose the “no trick” strategy, folks will choose the “exit” strategy, and if officials choose the “trick” strategy, folks will choose the “no exit” strategy to deal with it. Different from Scenario 1, even if the reputation loss caused by folks becoming nail households is lower than the expected cost of returning to poverty when officials adopt the “trick” strategy, as long as it is still higher than the expected cost of returning to poverty when officials adopt the “no tricks” strategy, the final ESS will still be the “no trick” strategy for officials and the “exit” strategy for folks.

##### *B, C, D* > 0, *A* < 0

There is only one ESS (1, 1) in the game, which needs to satisfy δ_2_*P*_2_ < *P*_3_ + *C*_*G*2_ + *P*_1_, δ_1_*P*_2_ > *C*_*G*2_ + *P*_1_, *E* > η_2_*R* > η_1_*R*. Similar to Scenario 1, the “exit” strategy is still the dominant strategy of folks. *A* < 0, *C* > 0 shows that if folks choose the “exit” strategy, officials will choose the “no trick” strategy, and if folks choose the “no exit” strategy, officials will choose the “trick” strategy to deal with it. Unlike Scenario 1, even facing the “no exit” strategy of folks, officials should choose the “trick” strategy to maximize their benefits. As folks continue to change from the “no exit” strategy to the “exit” strategy, officials will also change their coping methods to achieve an ideal ESS (1, 1).

##### *C, D* > 0, *A, B* < 0

**Figure 4:**
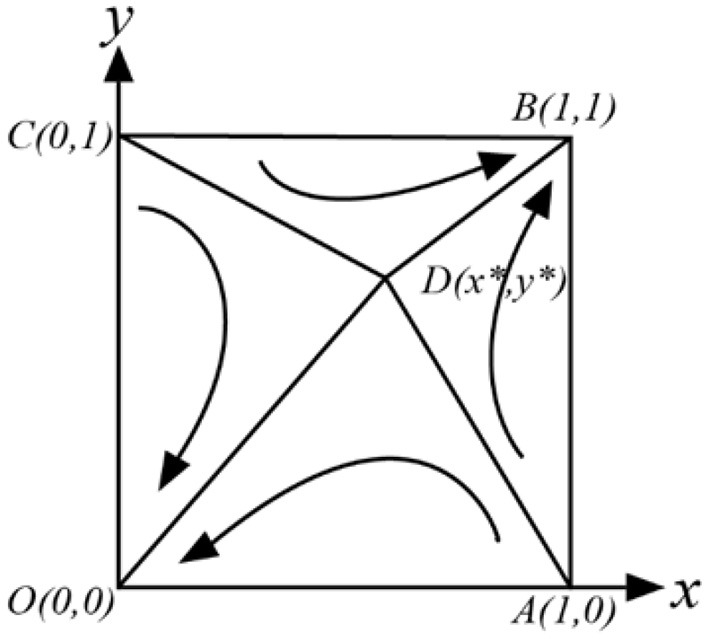
Evolutionary phase diagram of officials and folks.

There are two ESS (1, 1) or (0, 0) in the game, which need to satisfy δ_2_*P*_2_ < *P*_3_ + *C*_*G*2_ + *P*_1_, δ_1_*P*_2_ > *C*_*G*2_ + *P*_1_, η_2_*R* > *E* > η_1_*R*. In this case, neither officials nor folks have dominant strategies, and the final ESS of the game is related to the initial strategy selection ratio (*x, y*) of both parties. At this point, we can draw a phase diagram, as shown in Figure 4.

From Figure 4, we can see the relative size of the quadrilateral *S*_*ABCD*_ and *S*_*AOCD*_ determines the possibility of the game evolving to (0, 0) or (1, 1). To get the ideal ESS (1, 1), we need to increase the relative area of the quadrilateral *S*_*ABCD*_ as much as possible, where:


$$\begin{array}{l} \text{S}={\text{S}}_{\text{A}OC\text{D}}=\frac{1}{2}\left({\text{x}}^{{\;}^{*}}+{\text{y}}^{{\;}^{*}}\right) \end{array}$$

(14) $$\begin{array}{l} =\frac{1}{2}\left[\frac{\eta_{2}\text{R}-\text{E}}{\left(\eta_{2}-\eta_{1}\right)\text{R}}+\frac{{\text{P}}_{3}+{\text{C}}_{\text{G}2}+{\text{P}}_{1}-\delta_{2}{\text{P}}_{3}}{{\text{P}}_{3}+\delta_{1}{\text{P}}_{2}-\delta_{2}{\text{P}}_{3}}\right] \end{array}$$

Taking the partial derivative of each parameter in Equation (14), we can find that S is proportional to *E*, η_1_, *C*_*G*2_, *P*_1_, *P*_3_, and inversely proportional to *R*, δ_1_, δ_2_.

##### *A, B, D* > 0, *C* < 0

There is only one ESS (0, 1) in the game, which needs to satisfy δ_2_*P*_2_ > *P*_3_ + *C*_*G*2_ + *P*_1_, δ_1_*P*_2_ < *C*_*G*2_ + *P*_1_, *E* > η_2_*R* > η_1_*R*. In this case, the dominant strategy of folks is the “exit” strategy, at which the benefits of the officials who choose the “trick” strategy are higher than that of the “no trick” strategy, so ESS will eventually converge to (0, 1). If the government cannot adjust the game's parameters through punishment, reward, supervision, and so on., the ideal ESS (1, 1) cannot be reached in the end.

##### *B, D* > 0, *A, C* < 0

There is only one ESS (0, 1) in the game, which needs to satisfy δ_2_*P*_2_ < *P*_3_ + *C*_*G*2_ + *P*_1_, δ_1_*P*_2_ < *C*_*G*2_ + *P*_1_, *E* > η_2_*R* > η_1_*R*. In this case, the dominant strategy of the officials is the “trick” strategy. Still, the dominant strategy of folks is the “exit” strategy, so ESS will eventually converge to (0, 1).

##### *A, C* > 0, *B, D* < 0

There is only one ESS (1, 0) in the game, which needs to satisfy δ_2_*P*_2_ > *P*_3_ + *C*_*G*2_ + *P*_1_, δ_1_*P*_2_ > *C*_*G*2_ + *P*_1_,η_2_*R* > η_1_*R* > *E*. In this case, the dominant strategy of the officials is the “no trick” strategy. Still, the dominant strategy of folks is the “no exit” strategy, so ESS will eventually converge to (1, 0).

##### *A* > 0, *B, C, D* < 0

There is only one ESS (1, 0) in the game, which needs to satisfy δ_2_*P*_2_ > *P*_3_ + *C*_*G*2_ + *P*_1_, δ_1_*P*_2_ < *C*_*G*2_ + *P*_1_,η_2_*R* > η_1_*R* > *E*. In this case, the dominant strategy of the folks is the “no exit” strategy, in which the benefits of the “trick” strategy are lower than that of the “no trick” strategy, so ESS will eventually converge to (1, 0).

##### *D* > 0, *A, B, C* < 0

There is only one ESS (0, 0) in the game, which needs to satisfy δ_2_*P*_2_ < *P*_3_ + *C*_*G*2_ + *P*_1_, δ_1_*P*_2_ < *C*_*G*2_ + *P*_1_,η_2_*R* > *E* > η_1_*R*. In this case, the dominant strategy of the officials is “trick,” at which the benefits of the “exit” strategy are lower than that of the “no exit” strategy, so ESS will eventually converge to (0, 0).

##### *C* > 0, *A, B, D* < 0

There is only one ESS (0, 0) in the game, which needs to satisfy δ_2_*P*_2_ < *P*_3_ + *C*_*G*2_ + *P*_1_, δ_1_*P*_2_ > *C*_*G*2_ + *P*_1_,η_2_*R* > η_1_*R* > *E*. In this case, the dominant strategy of the folks is the “no exit” strategy, in which the benefits of the “trick” strategy are higher than that of the “no trick” strategy, so ESS will eventually converge to (0, 0).

##### *A, B, C, D* < 0

There is only one ESS (0, 0) in the game, which needs to satisfy δ_2_*P*_2_ < *P*_3_ + *C*_*G*2_ + *P*_1_, δ_1_*P*_2_ < *C*_*G*2_ + *P*_1_,η_2_*R* > η_1_*R* > *E*. In this case, the dominant strategy of the officials is the “trick” strategy. Still, the dominant strategy of folks is the “no exit” strategy, so ESS will eventually converge to (0, 0).

## Numerical Simulation and Discussion

The above analysis shows that the policy factors–poverty exit mechanisms including government's punishment, incentive, and supervision policies will affect the cognitive behaviors of officials and folks in the poverty exit work, making ESS converge to different equilibrium points. However, to achieve the win–win situation we expect, that is, (1, 1), the government needs to meet some limited parameters, such as: How severe should the punishment be? How strong should the incentive be? and How adequate should the supervision be?

Therefore, this study takes County Y of Province H as an example to carry out a numerical simulation. According to the actual situation of County Y, we assign reasonable values to the parameters in the game (representing the relative size of each parameter). Then, we simulate the evolution process of the official-folk game in the situations mentioned above and discuss the initial value of the ratio (*x, y*) of the strategies chosen by both parties of the game and the effect of policy parameters on ESS.

### Unfavorable Policy Parameters

#### Scenarios 9, 10, 11

**Figure 5:**
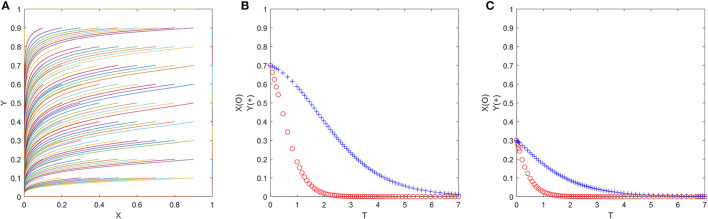
**(A–C)** The evolution process of the official-folk game under unfavorable policy parameters (Scenario 11).

Since the ESS of cases 9, 10, and 11 is all (0, 0), the numerical simulation results are also similar. Therefore, we only show the representative numerical simulation results of case 11 in Figure 5, where δ_1_ = 0.2, δ_2_ = 0.3, η_1_ = 0.3, η_2_ = 0.4, *P*_1_ = 3, *P*_2_ = 10, *P*_3_ = 2, *C*_*G*2_ = 1, *R* = 7, *E* = 2 (satisfy δ_2_*P*_2_ < *P*_3_ + *C*_*G*2_ + *P*_1_, δ_1_*P*_2_ < *C*_*G*2_ + *P*_1_, η_2_*R* > η_1_*R* > *E*). Figure 5A shows that no matter what the initial value of (*x, y*) is, ESS will eventually converge to (0, 0). Figures 5B,C are evolution trajectories at *x* = *y* = 0.7 and *x* = *y* = 0.3. The convergence speed of both sides of the game in (c) is significantly slower than (b), which indicates that the greater the probability that officials and folks are initially non-cooperative, the sooner they will fall into the prisoner's dilemma.

#### Scenarios 5, 6, 7, 8

**Figure 6:**
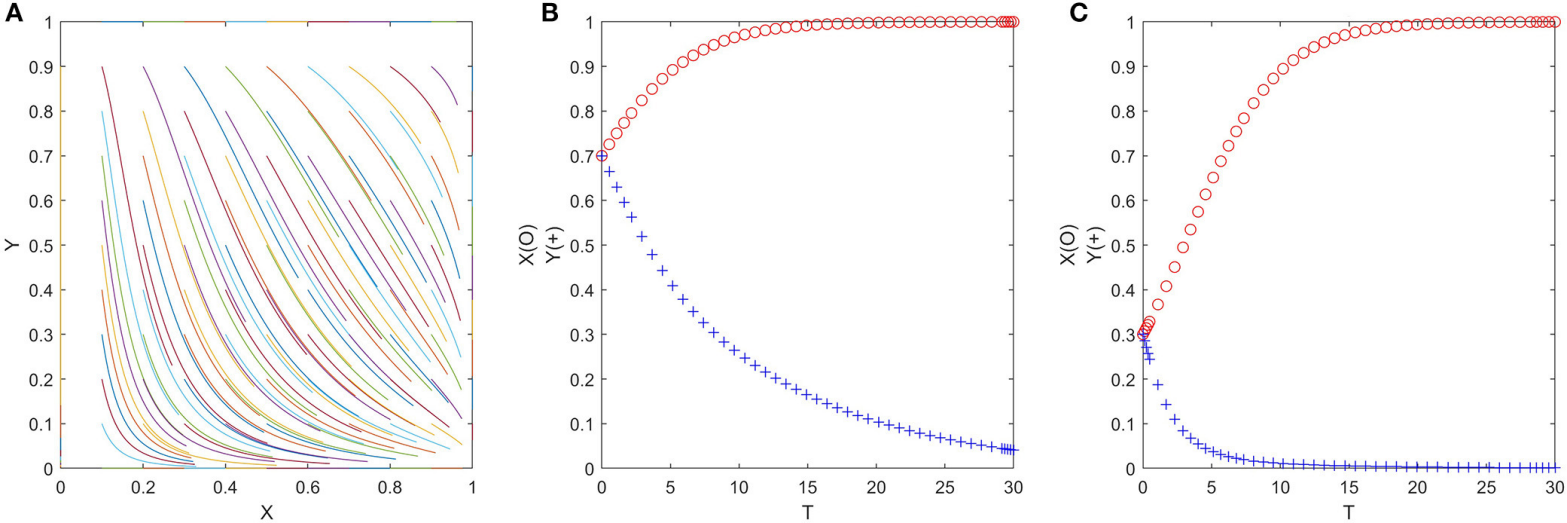
**(A–C)** The evolution process of the official-folk game under unfavorable policy parameters (Scenario 7).

Since the ESS of cases 5, 6, 7, and 8 is all (0, 1) or (1, 0), the numerical simulation results are also similar. Therefore, we only show the representative numerical simulation results of case 7 in Figure 6, where δ_1_ = 0.2, δ_2_ = 0.3, η_1_ = 0.3, η_2_ = 0.4, *P*_1_ = 3, *P*_2_ = 21, *P*_3_ = 2, *C*_*G*2_ = 1, *R* = 7, *E* = 2 (satisfy δ_2_*P*_2_ > *P*_3_ + *C*_*G*2_ + *P*_1_, δ_1_*P*_2_ > *C*_*G*2_ + *P*_1_, η_2_*R* > η_1_*R* > *E*). Figure 6A shows that no matter what the initial value of (*x, y*) is, ESS will eventually converge to (1, 0). Figures 6B,C are evolution trajectories at *x* = *y* = 0.7 and *x* = *y* = 0.3.

### Moderate Policy Parameters—Scenario 4

**Figure 7:**
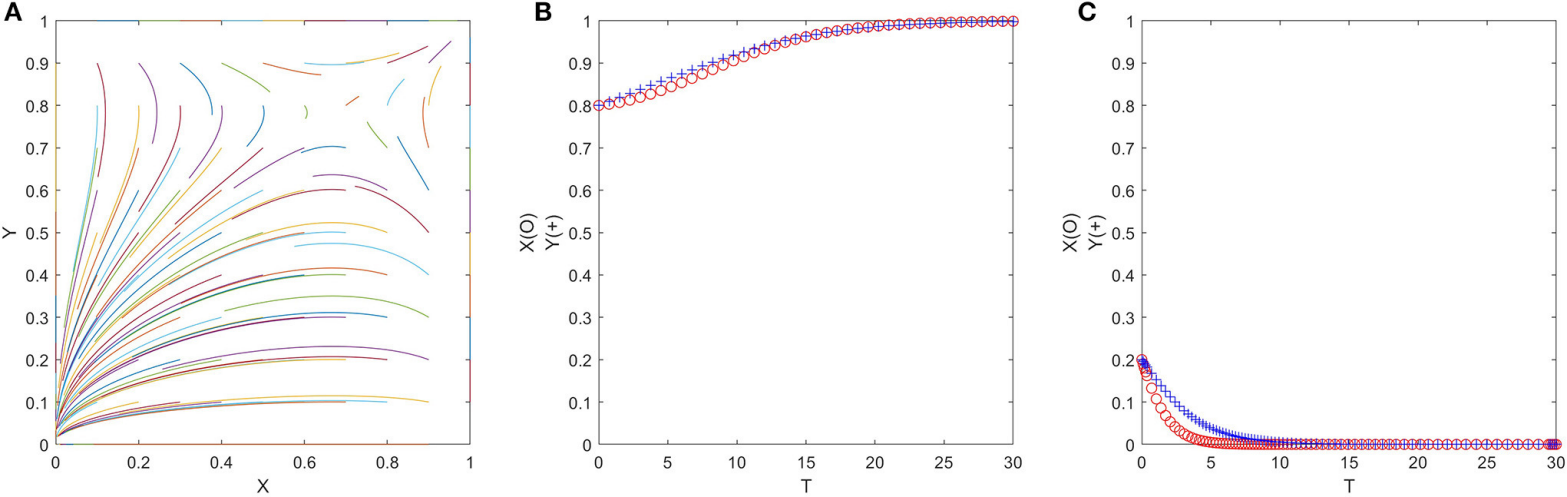
**(A–C)** The evolution process of the official-folk game under moderate policy parameters (Scenario 4).

The ESS of case 4 is (1, 1) and (0, 0), which one converges to is determined by the initial value of (*x, y*). We show the representative numerical simulation results of case 4 in Figure 7, where δ_1_ = 0.25, δ_2_ = 0.3, η_1_ = 0.3, η_2_ = 0.4, *P*_1_ = 3, *P*_2_ = 17, *P*_3_ = 2, *C*_*G*2_ = 1, *R* = 6, *E* = 2 (satisfy δ_2_*P*_2_ < *P*_3_ + *C*_*G*2_ + *P*_1_, δ_1_*P*_2_ > *C*_*G*2_ + *P*_1_, η_2_*R* > *E* > η_1_*R*), which shows that if officials and folks have a relatively high willingness to cooperate at the beginning and strictly follow the government's requirements in the poverty exit work (officials choose the “no trick” strategy, and folks choose the “exit” strategy), ESS will eventually converge to (1, 1). If officials and folks are hardly willing to cooperate at the beginning and only consider their interests, the ESS will eventually converge to (0, 0). However, the government can make the game more likely to reach an ideal ESS (1, 1) through external interventions (such as increasing the upper right quadrilateral *S*_*AOCD*_).

### Favorable Policy Parameters—Scenarios 1, 2, 3

**Figure 8:**
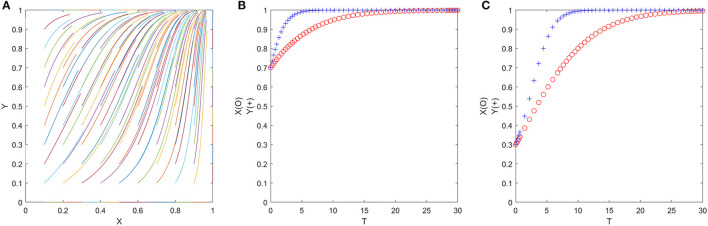
**(A–C)** The evolution process of the official-folk game under favorable policy parameters (Scenario 1).

Since the ESS of cases 1, 2, and 3 is all (1, 1), the numerical simulation results are also similar. Therefore, we only show the representative numerical simulation results of case 1 in Figure 8, where δ_1_ = 0.2, δ_2_ = 0.3, η_1_ = 0.3, η_2_ = 0.4, *P*_1_ = 3, *P*_2_ = 21, *P*_3_ = 2, *C*_*G*2_ = 1, *R* = 7, *E* = 3 (satisfy δ_2_*P*_2_ > *P*_3_ + *C*_*G*2_ + *P*_1_, δ_1_*P*_2_ > *C*_*G*2_ + *P*_1_, *E* > η_2_*R* > η_1_*R*). Figure 8A shows that no matter what the initial value of (*x, y*) is, ESS will eventually converge to (1, 1). In other words, as long as the policy adopted by the government meets the three conditions above, officials and folks can consistently achieve a win–win situation. Figures 8B,C are evolution trajectories at *x* = *y* = 0.7 and *x* = *y* = 0.3.

From the numerical simulation above, it can be found that neither of the two types of parameters shown in Figures 5, 6 can get officials and folks out of the prisoner's dilemma. Although the parameters shown in Figure 7 have the potential to allow both the government and the people to escape from the prisoner's dilemma and achieve a win–win situation, the final ESS depends on the initial ratio of both official and civilian populations, which significantly reduces the applicability of the model parameters. Therefore, we will focus on the favorable policy parameters (as shown in Figure 8) in the following.

The first parameter combination is δ_2_*P*_2_ > *P*_3_ + *C*_*G*2_ + *P*_1_, δ_1_*P*_2_ > *C*_*G*2_ + *P*_1_, *E* > η_2_*R* > η_1_*R* in Scenario 1, under which situation, regardless of the initial willingness to cooperate of the officials and folks parties, their willingness will gradually change in the game process. Finally, a win–win situation of cooperation will be achieved. To achieve this parameter combination, the government should appropriately increase δ_1_, δ_2_, and *P*_2_ and decrease *P*_1_, *P*_3_, η_1_, and η_2_. Specifically, the government can make efforts in the following aspects:

First, improve the reward-punishment mechanism in poverty alleviation and adhere to the principle of appropriateness while rewarding merit and punishing failures. Under the current political championships system, officials' performance has become the most critical promotion indicator, and exceeding the poverty alleviation indicators also has become the shared willingness of each grassroots cadre (Leng and Zuo, 2022). Besides, the “one-vote veto” system allows officials less interested in promotion to become a member of the tournament (Chang, 2018). In other words, the unreasonable reward-punishment mechanism gives officials the motivation and incentive to implement “Digital Poverty Alleviation” in targeted poverty alleviation. Therefore, establishing a perfect reward and punishment mechanism means that the government cannot combat officials' enthusiasm for poverty alleviation. At the same time, it must effectively weaken the problematic cognitive behavior of officials.

Second, a broader and more effective supervision mechanism for poverty alleviation and curb any illegal and chaotic behaviors was established. The reward-punishment mechanism and the supervision mechanism complement each other. No matter how good the reward-punishment mechanism is, it is not easy to achieve the desired effect without a suitable supervision mechanism to protect it (Liu et al., 2020). An appropriate monitoring mechanism should be not only top–down but also bottom–up (Serra, 2012). Although the government implements the investigation and punishment of various violations of regulations and disciplines through the State Council Poverty Alleviation Office, grassroots officials can always think of multiple ways to cope with the inspection and evaluation by taking advantage of information asymmetry (Liu et al., 2018). However, every citizen is a potential supervisor of poverty alleviation at the grassroots level. Suppose the government establishes a bottom–up supervision mechanism and feedback channel, allowing the whole people to supervise targeted poverty alleviation and poverty exit work. In that case, the problematic behavior of officials will be more effectively curbed.

Third, communication with folks was strengthen, and correct cognitive behavior was guided. Although many post-help policies have been promulgated to eliminate the folks' fear of returning to poverty, the results have not been evident (Zhang et al., 2022). Therefore, grassroots poverty alleviation cadres should conduct in-depth exchanges with poor households, keep abreast of the ideological trends of poor households, and adhere to the specific policy tailored to each household. For those poor households who fear returning to poverty, grassroots officials must do their ideological work and actively steer their cognitive behavior in the right direction. Officials must make folks understand the truth that they will still receive government support and assistance again while reencountering difficulties. For those poor households lifted out of poverty entirely but unwilling to give up preferential assistance conditions, poverty alleviation cadres should provide spiritual poverty alleviation to help them establish a sense of self-improvement to achieve well-off through their struggles.

The second parameter combination is δ_2_*P*_2_ > *P*_3_ + *C*_*G*2_ + *P*_1_, δ_1_*P*_2_ > *C*_*G*2_ + *P*_1_, η_2_*R* > *E* > η_1_*R* or δ_2_*P*_2_ < *P*_3_ + *C*_*G*2_ + *P*_1_, δ_1_*P*_2_ > *C*_*G*2_ + *P*_1_, *E* > η_2_*R* > η_1_*R* in Scenarios 2,3, under which situation, one of the officials or folks must compromise first and choose a strategy conducive to achieving win–win cooperation. After observing that the former decides to cooperate, the other party will cooperate; otherwise, it will not cooperate. There are two reasons for this situation: From the officials' view, even if his original intention is not to choose the “trick” strategy, he has nothing to do with those nail households who are determined not to choose the “exit” strategy. Coupled with the “one-vote veto” punishment mechanism, they must take a risk and choose the “trick” strategy. If the folks cooperate with the “exit” strategy, they will naturally choose the “no trick” strategy; from the perspective of the folks, although they worry about returning to poverty, most of them will still choose to cooperate with the “exit” strategy, if the officials patiently persuade them and inform them that taking off poverty hats does not mean lost policy help. However, if officials implement DPA, folks will find cooperating with officials difficult. Therefore, when the government formulates policies, it needs first to create a favorable situation in which one party will choose to cooperate no matter what. So that, driven by active partners, the other party will gradually cooperate and finally achieve a win–win situation.

Under the political system with Chinese characteristics, grassroots poverty alleviation cadres generally actively carry out targeted poverty alleviation work assigned by the government. However, grassroots poverty alleviation cadres often encounter all kinds of difficulties (both self and external) in the implementation process, so they often use their information advantages to create the illusion of poverty elimination (DPA) under the pressure of government assessment (Gao and Tyson, 2020). Since poor households are at a disadvantage in terms of power and information relative to grassroots poverty alleviation cadres, poor households who are weak in cadres' DPA will only take an attitude of letting it go. Therefore, poverty-stricken households who intended to remove poverty hats as required were reluctant to do so out of distrust of officials. Furthermore, after enjoying the benefits of the poverty alleviation policy, especially critical illness relief and medical assistance measures, some poor households are reluctant to give up these vested interests after they are lifted out of poverty. Even a “lazy effect” (Xu et al., 2021a) appears that poor households believe that they should enjoy the minimum living allowance and other poverty alleviation policies once they wear the poverty hats. Therefore, the cognitive-behavioral differences between officials and folks regarding the procedure, standards, and methods of removing the poverty hats form a new social dilemma called the official-folk game. Without the interference of exogenous policy factors, officials and folks will eventually get caught up in the prisoner's dilemma. Fortunately, the government can develop effective policies to counteract the effects of this social dilemma to some extent.

## Conclusion and Limitations

From the perspective of evolutionary game theory, this study analyzes the cognitive-behavioral differences between officials and folks and reveals their formation mechanism in China's targeted poverty alleviation, which has significant practical implications. The officials hope to achieve promotion and salary increases or not be “vetoed by one vote” through poverty exit work, thus motivating “Digital Poverty Alleviation.” The folks have relied on poverty alleviation policy and been unwilling to take off poverty hats out of distrust of officials and fear of returning to poverty. Without the intervention of exogenous factors, the result of the official-folk game between the officials and the folks will eventually lead to the prisoner's dilemma (i.e., officials choose the “trick” strategy, and folks choose the “no exit” strategy), which dramatically reduces the effect of the targeted poverty alleviation. In addition, this study also provides an effective solution to this social dilemma through an evolutionary game analysis, which indicates the direction for maximizing the effectiveness of targeted poverty alleviation. Furthermore, this finding also provides a reference for other developing countries to overcome similar social dilemmas in the process of eliminating absolute poverty. Ultimately, this finding points the way forward for the assistance encountered in alleviating relative poverty in the future.

Despite the valuable exploration of cognitive-behavioral differences between officials and folks in China's targeted poverty alleviation in this paper, there are still some shortcomings to be improved. First, the employed evolutionary game model is based on infinite and well-mixed populations, contrary to reality to a certain extent. We will consider a finite population, or the population has social network structures in the future. Second, we mentioned a new social dilemma (official-folk game) in this paper, but we did not quantify this dilemma's strength like Arefin et al. (2020) and Wang et al. (2015). In the future, we will find a method for our asymmetric binary game that quantifies the dilemma strength.

## Data Availability Statement

The original contributions presented in the study are included in the article/supplementary material, further inquiries can be directed to the corresponding author.

## Author Contributions

ZC contributed to the study's design, a compilation of interview records, and manuscript revision. CY contributed to numerical analysis and the manuscript edition. Both authors contributed to the article and approved the submitted version.

## Funding

This research was funded by the Hubei Provincial Social Science Foundation (Project #: 2021167).

## Conflict of Interest

The authors declare that the research was conducted in the absence of any commercial or financial relationships that could be construed as a potential conflict of interest.

## Publisher's Note

All claims expressed in this article are solely those of the authors and do not necessarily represent those of their affiliated organizations, or those of the publisher, the editors and the reviewers. Any product that may be evaluated in this article, or claim that may be made by its manufacturer, is not guaranteed or endorsed by the publisher.
